# Individual Aircraft Noise Exposure Assessment for a Case-Crossover Study in Switzerland †

**DOI:** 10.3390/ijerph17093011

**Published:** 2020-04-26

**Authors:** Apolline Saucy, Beat Schäffer, Louise Tangermann, Danielle Vienneau, Jean-Marc Wunderli, Martin Röösli

**Affiliations:** 1 Swiss Tropical and Public Health Institute (Swiss TPH), CH-4002 Basel, Switzerland; apolline.saucy@swisstph.ch (A.S.); louise.tangermann@swisstph.ch (L.T.); danielle.vienneau@swisstph.ch (D.V.); 2 Faculty of Science, University of Basel, CH-4003 Basel, Switzerland; 3 Empa, Swiss Federal Laboratories for Materials Science and Technology, CH-8600 Dübendorf, Switzerland; beat.schaeffer@empa.ch (B.S.); jean-marc.wunderli@empa.ch (J.-M.W.)

**Keywords:** exposure assessment, case-crossover, aircraft noise, cardiovascular diseases

## Abstract

Accurate exposure assessment is essential in environmental epidemiological studies. This is especially true for aircraft noise, which is characterized by a high spatial and temporal variation. We propose a method to assess individual aircraft noise exposure for a case-crossover study investigating the acute effects of aircraft noise on cardiovascular deaths. We identified all cases of cardiovascular death (24,886) occurring near Zürich airport, Switzerland, over fifteen years from the Swiss National Cohort. Outdoor noise exposure at the home address was calculated for the night preceding death and control nights using flight operations information from Zürich airport and noise footprints calculated for major aircraft types and air routes. We estimated three different noise metrics: mean sound pressure level (L_Aeq_), maximum sound pressure level (L_Amax_), and number above threshold 55 dB (NAT_55_) for different nighttime windows. Average nighttime aircraft noise levels were 45.2 dB, 64.6 dB, and 18.5 for L_Aeq_, L_Amax_, and NAT_55_ respectively. In this paper, we present a method to estimate individual aircraft noise exposure with high spatio-temporal resolution and a flexible choice of exposure events and metrics. This exposure assessment will be used in a case-crossover study investigating the acute effects of noise on health.

## 1. Introduction

Noise from road, railway and air traffic is one of the most widespread sources of environmental stress and discomfort in everyday life [1,2]. The impact of aircraft noise on health has been increasingly recognized—especially in relation to long-term annoyance, sleep disturbance, and cardiovascular health outcomes. For instance, the Swiss Government recently established a national plan aiming to limit noise at source to promote population health, especially in the urban environment [3]. The Swiss Noise Abatement Ordinance of 1986 defines exposure limits for traffic noise and other technical noise sources. It limits permissible emissions at the source and contains building restrictions for areas exceeding the noise limits [4]. The World Health Organization (WHO) recently released new guidelines recommending that the average nighttime exposure to aircraft noise should stay below 40 dB [5]. A previous study conducted in the Swiss population reported an increased risk of death from myocardial infarction associated with long-term exposure to traffic noise. For an increase of 10 dB L_den_ (day-evening-night level, where evening levels get a 5 dB and night a 10 dB penalty), the hazard ratios were 1.04 (95% confidence interval: 1.02–1.06), 1.02 (1.01–1.03), and 1.03 (1.01–1.05) for road traffic, railway, and aircraft noise, respectively [6]. Aircraft noise has also been shown to be associated with increased risk of hypertension, cardiovascular diseases and hospital admissions [7,8,9]. For ischemic heart disease, the recent WHO environmental noise guideline reports a risk ratio of 1.09 (1.04–1.15) per 10 dB L_den_ increase in aircraft noise [5].

While experimental studies tend to increasingly draw attention to the short-term effects of noise—including aircraft noise—on sleep disturbance [10,11], blood pressure [12,13], glucose and other metabolic perturbations [2,14], most of the existing epidemiological studies investigating the effects of noise on mortality focus on chronic noise exposure [1,6,15]; and thus less is known about the acute effects of transportation noise on cardiovascular mortality. It is particularly important to consider the timing of noise exposure when investigating the acute effects of transportation noise on health, including potential physiological differences in the different sleep phases during the night [2], as well as possible differences in the effects of transportation noise on sleep and mortality during various parts of the night [11,16]. In this regard, the daily variations in flight schedules and routes present in many airports offer an appealing opportunity to conduct case-crossover studies to investigate the acute effects of aircraft noise on mortality.

In addition to the question of timing, particular attention should be paid to environmental noise characteristics and metrics. Noise exposure is complex, with high temporal and spectral variation, where a simple estimate of the daily mean might lead to a loss of important components of noise characteristics when investigating short term effects [17]. This potential source of error or misclassification can have consequences on the observed physiological response, which in turn will reduce explained variance. For instance, Héritier et al. [6] showed that novel exposure metrics such as the intermittency ratio could account for temporal variations observed between different sources of traffic noise. Another recent study highlighted the importance of several noise metric combinations and the number of events to account for the observed annoyance associated with aircraft noise exposure [18]. In order to investigate the individual role of various nighttime exposure windows and metrics, a reliable and detailed noise exposure assessment is required.

The aim of this paper is to describe a methodology to calculate individual aircraft noise exposures for various time windows, required to conduct a case-crossover study investing effects of aircraft noise on myocardial infarction, stroke and other ischemic cardiovascular causes of mortality, in the framework of the TraNQuIL (Transportation Noise: Quantitative Methods for Investigating Acute and Long Term Health Effects) project. We propose a method to calculate several noise metrics that can be used individually and combined. This paper is an extended version of our conference proceedings published in [19].

## 2. Materials and Methods

### 2.1. Case-Crossover Design

**Figure 1:**
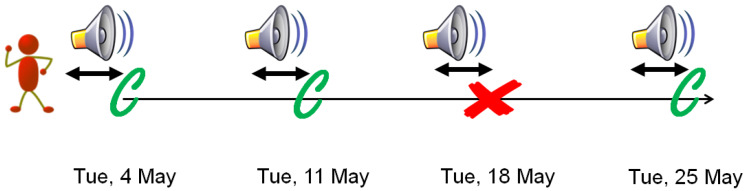
Example of case-crossover design, where exposure (noise level) is assessed in case (red) and control (green) event nights for an individual.

A case-crossover study is designed to investigate acute health effects from time-varying exposures such as air pollution, physical activity, emotional stress, or noise [20,21]. Analogous to a case-control study, the underlying question is how unusual the exposure situation is when an event occurs (case events) compared to the typical exposure when no event occurred (control events). Thus, exposure levels for case events are compared with exposure levels for control events as presented in Figure 1. It is a case-only study design with the advantage that it is not vulnerable to confounding from individual characteristics that are generally stable over a short period of time, such as age, gender or lifestyle factors [20]. Adjustment is typically required for a series of time-varying variables, such as air pollution or meteorological conditions. Since the first description of the case-crossover design by Maclure in 1991 [21], the framework has been commonly used to investigate the acute effects of various behavioral exposures, such as coffee intake or physical activity [20,22]. More recently, it has been increasingly applied to environmental exposures—mainly air pollution, but also wind turbine noise [23,24]. The case-crossover design is very well suited to investigate environmental exposures, given sufficient temporal variation in exposure. Due to its extensive application in air pollution studies, potential bias and sampling strategies are well documented in this context [25,26]. In brief, the case-crossover framework is proposed as an alternative to time-series and data can be analyzed using conditional logistic regression. As future environmental exposures are typically not influenced by the event status (for instance hospitalization or death), control events should be selected both before and after the event to reduce the risk of bias due to time trends in the exposure time-series [27,28,29]. We propose to apply the same approach to investigate the acute effects of aircraft noise on mortality. At Zürich Airport (ZRH), meteorological conditions influence the daily flight schemes, offering day-to-day variability in individual noise exposure levels. As air operations may show weekly variation, we chose a time-stratified control sampling approach, where control events are matched on the day of the week within the same month, leading to 3–4 selected control events per case event, as described by Carracedo-Martínez et al. [23].

### 2.2. Zürich Airport

**Figure 2:**
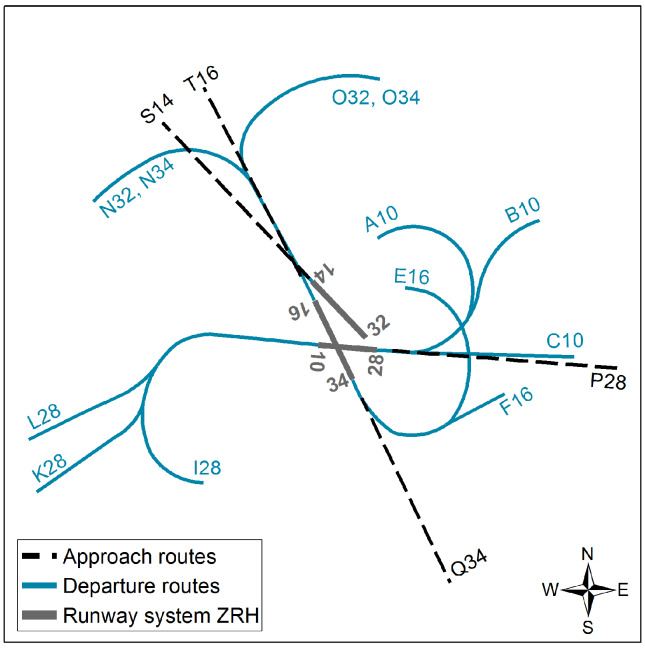
Overview of the runway system and air routes at Zürich Airport (ZRH).

Zürich Airport (ZRH) is the largest airport in Switzerland in terms of air traffic. It is composed of a system of three runways, offering 12 major departure and four approach routes for commercial air traffic (see Figure 2). The assignment of air traffic to routes can change from day to day depending on different factors such as wind direction. Therefore, noise exposure at a given location is expected to vary between case and control days [30]. ZRH is subject to a flight ban, which limits the flight traffic to permitted exceptions such as emergency flights. The flight ban was set from 00:30 to 05:00 (approaches) and 06:00 (departures) in 2000 and extended to 23:30 to 06:00 in 2010 [31].

### 2.3. Study Population

**Figure 3:**
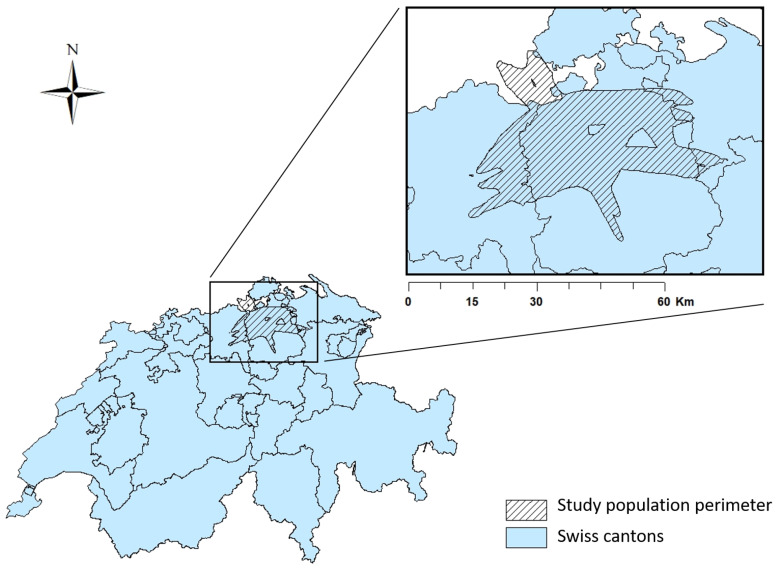
Overview of the study area used to select the study population around ZRH.

The study population was selected from the Swiss National Cohort (SNC) [32] in the vicinity of ZRH. It includes all individuals aged more than 30 years, dying from a cardiovascular cause (ICD10 classification I0 to I99) between 2000 and 2015. Only individuals potentially exposed to relevant aircraft noise exposure levels were selected. For this purpose, we used the envelope of the calculation perimeters for the Zürich Aircraft Noise Index (ZFI), which is a noise effect index for the number of highly annoyed and highly sleep disturbed persons (minimum L_Aeq_ of 47 dB during the day and/or 37 dB during the night) [33] (see Figure 3).

Geocoded residence at time of death were available from the SNC, together with other relevant personal information such as cause and time of death [32,34].

The use of the SNC data for this study was approved by the cantonal ethics boards of Bern (KEK No 205/06) and Zürich (KEK No 13/06).

### 2.4. Noise Exposure Assessment

Individual exposure was determined at the home location for the night before death and for the control nights, within the same month. Only nighttime exposure to aircraft noise was assessed, focusing the investigation on the effects of noise on mortality during sleeping phases. In addition, home exposure is expected to represent the effective exposure more accurately during nighttime than daytime, as people are more likely to be at home. We calculated three different metrics for nighttime aircraft noise: (1) the equivalent continuous sound pressure level (L_Aeq_) (2) the mean A-weighted and slow-time-weighted maximal event level (L_Amax_) and (3) the Number Above Threshold 55 dB (NAT_55_). These three exposure metrics, used both individually and combined, were chosen to represent the energetic and intermittent characteristics of aircraft noise [18].

Two separate approaches were considered for death cases occurring during the night and cases occurring during the day. For individuals dying during the day (07:00–23:00), we considered different exposure windows in the night preceding death, which roughly represents sleeping behaviors at the population level—such as the hours when individuals typically fall asleep, are asleep (core night), and wake-up from sleep (early morning)—as used in previous studies investigating the chronic effects of noise on health [10,16]. In addition, the selected time windows are representative of the particular flight situation present at ZRH, such as the reduced air traffic period and the nighttime flight ban (see Table 1). For people dying during the night (23:00–07:00), noise exposure was calculated for the two hours preceding the death, in order to investigate potential triggering effects of noise within 2 h, as described for other exposures [22]. The different exposure windows for daytime and nighttime deaths are listed in Table 1. Case and control events were created for all selected case and control dates and their respective exposure windows, separately for daytime and nighttime deaths.

Lists of movements are available for 2000 to 2015 and include detailed information for all aircraft departures and arrivals at ZRH, such as aircraft type, air route, runway and time of departure or landing. The departure or landing time is defined as the moment of aircraft touch down or brake release. An additional 10 min buffer was added before landing times and after departure times to account for the moment when the aircraft was perceived by the study population more distant from the airport. Some flights have missing information for the aircraft type and/or the air route. Using the tail number of the aircraft and the date of the event, missing aircraft types were retrieved. We selected only large aircraft types (>8618 Kg), as air traffic of small aircraft is negligible during the night.

As acoustic input, we used so-called footprints of aircraft noise events, previously calculated on a yearly basis at the authors’ institution, Empa [35]. A footprint corresponds to a 250 m receiver grid of mean noise exposure levels per aircraft type and air route. Each footprint is specific for a certain year, aircraft type (or group of aircraft types with similar flight performances), procedure (departure or arrival), air route, and possibly the time of day (e.g., day, night). Calculations were done with the aircraft noise calculation program FLULA2 [35] using individual flight trajectories as obtained from large radar data sets [30]. FLULA2 considers sound source data (sound emission level and directivity patterns) of individual aircraft types, numbers, and distributions of movements, detailed flight geometries, and topography. FLULA2 calculations represent standard atmospheric conditions [36]. From the level-time-histories LA(t) of the individual flights, the L_Amax_ and sound exposure level L_AE_ (resulting in the total energy of an event) are calculated, from which indicators such as the L_Aeq_ or the L_den_ could be derived. As a result of the calculations, the above-mentioned noise footprints (L_AE_ and L_Amax_) were stored.

All flights occurring during the previously described time windows were selected and joined to their respective case and control events. Using information on year, time, aircraft type, air route, and procedure contained within the list of movements, the respective footprints were identified. Each of the identified footprints—a footprint represents the average noise exposure for a number of flights of a certain aircraft type (or aircraft group) on a specific air route—were individually imported to collect the noise metrics of interest. The process was repeated for each footprint, so that each identified flight was associated with eight noise exposure values (4 nearest L_AE_ and L_Amax_). In a situation where no footprint was found, it was replaced by a similar footprint from a different time or year.

For each flight event, the average L_AE_ and L_Amax_ at the residential geocode was calculated from the four nearest noise receiver grid points using Inverse Distance Weighting (1).

(1) $$\begin{array}{c} \begin{array}{c} f\left(d\right)=\left\{\begin{array}{c} d_{i}>0,L=\frac{\sum_{i=1}^{4}\left(L_{i}*\frac{1}{d_{i}}\right)}{\sum_{i=1}^{4}\left(\frac{1}{d_{i}}\right)}\\ d_{i,min}=0,L=L_{i} \end{array}\right\}\\ d_{i}=\mathrm{distance}\;\mathrm{to}\;\mathrm{neighbour}\;i\\ L=\mathrm{Noise}\;\mathrm{metric}\;(L_{\mathrm{AE}}\;\mathrm{or}\;L_{\mathrm{Amax}})\\ L_{i}=\mathrm{Noise}\;\mathrm{level}\;\mathrm{at}\;\mathrm{residential}\;\mathrm{geocode}\;i \end{array} \end{array}$$

For L_AE_, the averaged noise levels of all events were energetically summed for case and control events exposure time windows (2).

(2) $$\begin{array}{c} \begin{array}{c} LAE_{i}=\sum_{i=1}^{n}\left(LAE_{i}\right)=10*\log\sum_{i=1}^{n}\left(10^{\frac{{L_{\mathrm{AE}}}_{i}}{10}}\right)\\ i=\mathrm{flight}\;\mathrm{event}\;i\\ n=\mathrm{number}\;\mathrm{of}\;\mathrm{flight}\;\mathrm{events}\;\mathrm{for}\;\mathrm{each}\;\mathrm{case}\;\mathrm{and}\;\mathrm{control}\;\mathrm{event}\;\mathrm{and}\;\mathrm{each}\;\mathrm{time}\;\mathrm{window} \end{array} \end{array}$$

Finally, the L_Aeq_ were calculated for the different time windows (see Equation (3)). The case and control events for which no flight was found or the final L_Aeq_ values were negative were set to zero dB.

(3) $$\begin{array}{c} \begin{array}{c} L_{\mathrm{Aeq}}=L_{\mathrm{AE}}-10*\log\left(\frac{T}{t_{0}}\right)\\ T=\mathrm{time}\;\mathrm{within}\;\mathrm{each}\;\mathrm{exposure}\;\mathrm{time}-\mathrm{window}\;\left[\mathrm{second}\right]\\ t_{0}=1\;s \end{array} \end{array}$$

**Figure 4:**
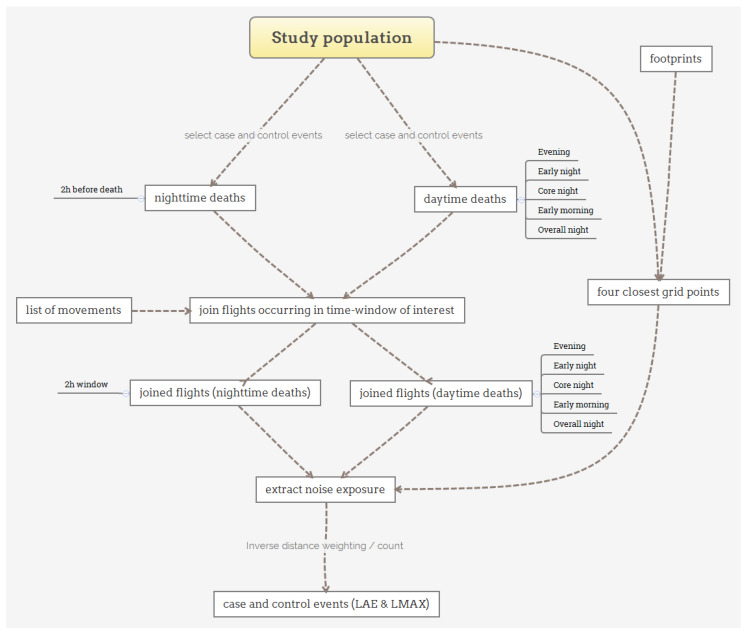
Graphical overview of the noise exposure assessment procedure.

For L_Amax_, the highest level of L_Amax_ observed within each case and control event window was defined as the maximum noise level. Additionally, the number of flights with a L_Amax_ value larger than 55 dB was counted, giving the Number Above Threshold, NAT_55_. The different steps of noise exposure assessment are illustrated in Figure 4.

## 3. Results

The above-described process resulted in the creation of a database listing individual aircraft noise exposure metrics (L_Aeq_, L_Amax_, and NAT_55_) for each case and control event and time window of interest. Below, we give some exemplary results as calculated for our study population.

Overall, 4,664,132 flights started or landed at ZRH between 2000 and 2015. Only 216 flights were excluded because of missing air route information. Selecting only large aircraft starting or landing during the hours of interest (18:50–07:10) reduced the data to 1,124,748 flights.

**Figure 5:**
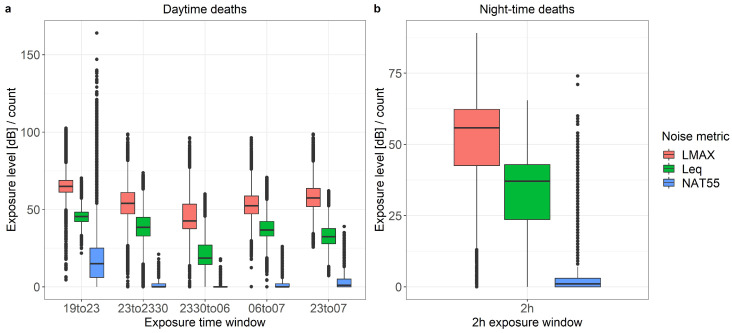
(**a**) Distribution of the noise exposure levels L_Amax_ and L_Aeq_ (in dB) as well as NAT_55_ (count) for the different time windows among all events (case and control) for daytime deaths, years 2000–2015. (**b**) Distribution of the noise exposure levels L_Amax_, L_Aeq_ and NAT_55_ for the 2 h exposure window among the events (case and control) for nighttime deaths, years 2000–2015. The horizontal line of the box-plot represents the median value, the squares the interquartile range (IQR), and the whiskers the lower and upper limits (lower IQR value—1.5*IQR/upper IQR value + 1.5*IQR).

Figure 5 shows the distribution of the L_Aeq_, L_Amax_ and NAT_55_ exposure levels for 24,886 cases and 84,597 control events by time window, separately for day and night death events. For daytime deaths (Figure 5a), exposure was highest for the evening exposure window (19:00–23:00) and lowest during the core night (23:30–06:00) as expected for all three exposure metrics. Median L_Aeq_ of the different time windows ranged from 20 to 45 dB (max. 75 dB) and L_Amax_ median values from 40 to 60 dB (max. 100 dB). NAT_55_ ranged between 0 and 20 during the core night and between 0 and 160 for the evening exposure window. For the nighttime deaths (Figure 5b), median L_Aeq_(2 h) was 36 dB with a maximum value about 65 dB and the average L_Amax_ was 57 dB with events up to 85 dB. The median NAT_55_ ranged between 0 and 75 flights for the 2 h exposure window preceding the time of case and control events.

## 4. Discussion

Noise is a transient and quickly evolving exposure, which makes it different from other environmental exposures. Aircraft noise typically presents more variation over time and according to WHO, the cardiovascular effects associated with aircraft noise exposure are also weaker than for road traffic noise [15]. Therefore, it is particularly important to limit potential exposure misclassification. Accurate exposure assessment is needed to better understand the role of different noise characteristics and the timing of exposure on health outcomes.

In order to tackle these issues, we developed a method to assess individual aircraft noise exposures with a high temporal and spatial resolution to support a case-crossover epidemiological study design. We illustrate examples of exposure estimates for specific time windows within a selected population around ZRH having died from cardiovascular disease during 2000–2015, to be used in further epidemiological health studies. It uses a list of movements from ZRH and links them with previously calculated aircraft noise footprints for different aircraft types and air routes at various points in time. These calculations are based on validated simulations, using individual flight trajectories and radar data, and take into account the general topography. With this method, we could recreate individual aircraft noise exposure for a large population sample over a period of 2000 to 2015 and extract three different noise metrics to investigate and describe potential short-term health effects in further studies.

The novelty of the approach proposed here relies on the combination of using a case-crossover design to investigate the possible effects of aircraft noise on health and detailed aircraft noise calculation available for our study population. The case-crossover design is particularly well suited to investigate aircraft noise, as flight patterns around airports with a multi-directional runaway system vary from day to day, offering sufficient exposure variation. The choice of exposure events is very flexible and precise, which makes this an attractive approach for conducting case-crossover studies investigating short-term or transient effects of noise on health. This framework accounts for several potential individual confounders and reduces the risk of bias resulting from many individual characteristics. It is, however, more sensitive to time-varying exposures, such as air pollution and meteorological factors, which need to be adjusted for in further epidemiological studies. The case-crossover design can also be quite sensitive to the selection of control events and can potentially have an impact on temporal bias and overall power. When applied to environmental exposures, a bi-directional control sampling approach—like the time-stratified sampling scheme chosen in the present study—together with a choice of control referents matching the most important time-varying factors, enable to reduce temporal bias [25]. In the present paper, we propose a sampling scheme matched on the day of the week due to expected weekly variations in the flight schemes and health events. High data quality makes our exposure assessment precise, although some exposure misclassification may occur if people are not at home during the night. This would produce an underestimation of a true risk but not a false positive result if there were no association. Other individual varying factors, such as alcohol intake or physical exercise cannot always be taken into account in this retrospective cohort setting. Nevertheless, due to its differences towards other existing studies in the field—including in terms of strengths and limitations—this approach is likely to offer meaningful insights in our general understanding of the association between aircraft noise and mortality. It also offers the possibility to investigate several noise metrics and their possible combinations to improve our understanding of the relationship between aircraft noise and mortality. The aircraft noise footprints used in the present approach are specific for our study area. However, lists of movements should be easily available in other locations. The proposed method can be adapted and applied to many different settings and used as a precedent to assess individual aircraft noise exposure based on lists of airports’ flight events.

## 5. Conclusions

We present a method to assess individual aircraft noise exposures with high temporal and spatial resolution. This method, especially designed to support a case-crossover study, represents a novel framework to investigate the short-term effects of aircraft noise on mortality. We propose to apply this approach to retrospective data and this paper may, therefore, serve as an exposure assessment method in large, long-term cohort settings. Due to its differences towards other study designs in terms of possible bias and confounding, this approach may complement previous research and bring meaningful insights in our general understanding of the acute physiological effects of noise.

## Figures and Tables

**Table 1:** List of the five different nighttime exposure windows considered for death case events occurring during the day and the night separately.

Exposure Time Window	Description	Daytime Deaths	Nighttime Deaths
		07:00 < 23:00	23:00 < 07:00
19:00 < 23:00	Evening	X	
23:00 < 23:30	Early night (reduced air traffic) *	X	
23:30 < 06:00	Core night (flight ban)	X	
06:00 < 07:00	Early morning	X	
23:00 < 07:00	Overall night	X	
2 h	2 h preceding time of death		X

* Reserved for delayed flights.

## Abbreviations

The following abbreviations are used in this manuscript:

L_Aeq_	A-weighted equivalent continuous sound pressure level over a defined period of time
L_AE_	A-weighted total energy of an event condensed on one second [dB]
L_Amax_	A-weighted maximum reached energy level of an event [dB]
L_den_	Day-evening-night level
NAT_55_	Number of events with L_Amax_ exceeding a threshold of 55 dB
SNC	Swiss National Cohort
WHO	World Health Organization
ZFI	Zürich Aircraft Noise Index
ZRH	Zürich Airport
